# Repurposing the Killing Machine: Non-canonical Roles of the Cell Death Apparatus in *Caenorhabditis elegans* Neurons

**DOI:** 10.3389/fcell.2022.825124

**Published:** 2022-02-14

**Authors:** Karen Juanez, Piya Ghose

**Affiliations:** Department of Biology, The University of Texas at Arlington, Arlington, TX, United States

**Keywords:** neuron, apoptosis, engulfment, cell division, regeneration, localized elimination, pruning

## Abstract

Here we highlight the increasingly divergent functions of the *Caenorhabditis elegans* cell elimination genes in the nervous system, beyond their well-documented roles in cell dismantling and removal. We describe relevant background on the *C. elegans* nervous system together with the apoptotic cell death and engulfment pathways, highlighting pioneering work in *C. elegans*. We discuss in detail the unexpected, atypical roles of cell elimination genes in various aspects of neuronal development, response and function. This includes the regulation of cell division, pruning, axon regeneration, and behavioral outputs. We share our outlook on expanding our thinking as to what cell elimination genes can do and noting their versatility. We speculate on the existence of novel genes downstream and upstream of the canonical cell death pathways relevant to neuronal biology. We also propose future directions emphasizing the exploration of the roles of cell death genes in pruning and guidance during embryonic development.

## 1 Introduction

Since the selection of *Caenorhabditis elegans* (*C. elegans*) as a model system by Sydney Brenner (Brenner 1973) and the subsequent completion of the first wiring diagram mapping all neurons (White et al., 1986), the *C. elegans* system has emerged seemingly tailored to study nervous system function and development. Great strides have been made, including to our understanding of animal behavior (Schafer 2005), neuronal repair (El Bejjani and Hammarlund 2012), neurite outgrowth (Jin and Kim 2020) and guidance (Chisholm et al., 2016). Studies of the nematode nervous system remain highly relevant with a recent focus that includes neuropil organization (Rapti et al., 2017; Moyle et al., 2021), connectome comparisons (Witvliet et al., 2021) with the worm’s brain and the molecular topography of the entire nervous system (Yemini et al., 2021) among other fascinating arenas.

Research in the *C. elegans* model system also pioneered genetic studies on programmed cell death (Yuan et al., 1993; Shaham 1998; Horvitz 2003; Geng et al., 2009), a crucial event for proper development and homeostasis. This work established that death can be programmed and dictated by specific genes. An entire pathway was elucidated, and these genes were found to be conserved across species up to vertebrates. While the classical view was that the activation of the canonical cell death pathway leads to the destruction of the entire cell through a form of programmed cell death called apoptosis, in the last decade, the roles of “cell death genes” have expanded to other contexts. These include both regressive events such as selective elimination of a part of a cell and restorative events such as axon repair. The aim of this review is to highlight novel non-canonical roles of classical cell death genes in neurons (Table 1) and to speculate on where the new horizon lies for these genes in the nervous system. We present pertinent background on the nematode nervous system as well as the core cell death pathways. We describe work demonstrating divergent roles of cell elimination genes in asymmetric cell division of neuronal precursor cells, neuronal function, as well as restorative (regeneration) and destructive (for example, pruning) events in the nervous system. We end by providing an outlook and propose further investigation during embryonic neuronal development.

**TABLE 1:** Summary of non-canonical roles of apoptotic death and engulfment genes in *C. elegans* neurons.

Non-apoptotic function		*C. elegans* cells/neurons	Cell death regulator	Mechanism	References
Asymmetric cell division		NSMnb	CED-3/caspase	Involves canonical apoptotic pathway upstream regulators. CED-1/MEGF10-dependent CED-3/caspase gradient	Chakraborty et al. (2015), Mishra et al. (2018)
		Q neuroblast	CED-3/caspase	Involves canonical apoptotic pathway upstream regulators. PIG-1/MELK dependent gradient of mitotic potential antagonized by CED-3/caspase	
Neuronal function	Glutamatergic behavior	GLR-1 expressing neurons	CES-1/Snail-like	Bidirectional regulation of glutamatergic signaling cell autonomously	Park et al. (2021)
	Sleep	ALA	CEP-1/p53	Activation following stressed-induced EGF signaling; promotes transcription of *egl-1*/BH3-only	DeBardeleben et al. (2017)
	Neuronal activity	Primarily URX	EGL-1/BH3-only	*egl-1* expression requires sensory transduction; translation and role in URX-related phenotypes unknown	Cohn et al. (2019)
Axon Regeneration	Early	ALM	CED-3/caspase	Promotes regeneration. EGL-1/BH3-only and CED-9/Bcl-2. independent. CED-3/caspase acts downstream of calcium/CRT-1/Calreticulin and upstream of DLK-1	Pinan-Lucarre et al. (2012)
			CED-4/Apaf-1		
	Initiation	PLM and D type motor neurons	CED-4/Apaf-1	Local activation of CED-4/Apaf-1 by CRT-1/Calreticulin/Calcium leads to CED-3/caspase activation and CED-7/ABC transporter cleavage resulting in PtdSer exposure. This “save me signal” is recognized by INA-1/PAT-3 which signals through CED-2/CrkII-CED-5/DOCK180-CED-12/ELMO to activate CED-10/Rac GTPase	Neumann et al. (2015), Hisamoto et al. (2018)
			CED-3/caspase		
			CED-7/ABC transporter		
			PtdSer		
			TTR-11/transthyretin		
			INA-1/PAT-3		
			CED-2/CrkII-CED-5/DOCK180-CED-12/ELMO		
			CED-10/Rac GTPase		
	Late	PLM	PtdSer	PSR-1 functions cell autonomously and parallel to its canonical engulfment pathway	Wang et al. (2010), Neumann et al. (2015), Abay et al. (2017)
			PSR-1	EFF-1 accumulates at cut site of proximal and distal membrane, facilitating fusion	
			CED-7/ABC transporter		
			TTR-52/transthyretin		
			NRF-5		
			CED-6/GULP		
			EFF-1		
Non-apoptotic death	Necrosis	GABA motor neurons	CED-4/Apaf-1	Mitochondrial CoQ-depletion may lead to CRT-1/calreticulin-dependent calcium release to activate CED-4/Apaf-1 leading to necrosis	Earls et al. (2010)
Localized Elimination	Synapse Elimination	RME + DD motor neurons	Canonical apoptotic pathway	Cell autonomous apoptotic pathway is activated by axonal mitochondria in synaptic regions. GSNL-1 is activated by CED-3/caspase which then severs actin filaments	Meng et al. (2015)
	Axon debris clearance	PLM	CED-1/MEGF10	CED-1/MEGF10 and CED-6/GULP act non-cell autonomously in surrounding epidermis/hypodermis	Nichols et al. (2016)
			CED-6/GULP	CED-7/ABC transporter and NRF-5 both promote and suppress axon degeneration during development	
			CED-2/CrkII-CED-5/DOCK180-CED-12/ELMO		
			CED-10/Rac GTPase		
			CED-7/ABC transporter		
			NRF-5		
	Axon regeneration and debris removal	ALM	CED-1/MEGF10	Two separate functions of CED-1/MEGF10; in axonal debris removal within engulfing muscle cell and axonal regeneration	Chiu et al. (2018)
			CED-6/GULP	CED-6/GULP downregulates CED-1/MEGF10 to inhibit axon regrowth	
	Pruning by glia	AFD	TAT-1/ATP8A	Temperature-dependent inhibition of PtdSer exposure by TAT-1/ATP8A. Selective engulfment following PtdSer recognition by PSR-1 and PAT-2/a-integrin resulting in activation of CED-2/CrkII-CED-5/DOCK180-CED-12/ELMO and CED-10/Rac GTPase	Raiders et al. (2021)
			PtdSer		
			PSR-1		
			CED-2/CrkII-CED-5/DOCK180-CED-12/ELMO		
			CED-10/Rac GTPase		
			PAT-2/a-integrin		
	Compartmentalized Cell Elimination (CCE)	Tail-Spike Cell, CEM neuron	CED-3/caspase	EGL-1/BH3-only-independent death. CED-3/caspase may have independent function in each cell compartment	Schwartz and Horvitz (2007), Ghose et al. (2018), Ghose and Shaham (2020)
			EFF-1	EFF-1seals phagosome during process elimination. Acts in engulfing cell	

### 1.1 The Nematode *Caenorhabditis elegans* and Its Nervous System

*C. elegans* achieved model organism status when Sydney Brenner selected the free-living soil nematode to study animal behavior and development (Brenner 1973). *C. elegans* has since established itself as a powerful genetic and cell biological tool with numerous advantages (Rapti 2020). Importantly, the anatomy of the animal is simple, with less than 1000 somatic cells in hermaphroditic adults. These include 302 neurons in the hermaphrodite nervous system. Despite a relatively simple nervous system, *C. elegans* exhibit several complex and well-defined behaviors including, locomotion, roaming and dwelling, foraging, feeding, mate searching and quiescence (Flavell et al., 2020).

Among many pioneering studies, the *C. elegans* nervous system was the first to be reconstructed to the level of the synapse. The complete circuitry and pattern of synaptic connectivity of this simple nervous system was determined by serial section electron microscopy (White et al., 1986). And with that, the stage was set to determine the genes involved in *C. elegans* neuronal development and neuronal function. The 302 neurons of the *C. elegans* hermaphrodite nervous system have their cell bodies largely organized in head or tail ganglia. Most neurons are thin (typically 100–200 nm in diameter) unipolar or bipolar with mainly unbranched processes or neurites. Neurons communicate via chemical synapses, gap junctions and neuromuscular junctions (NMJs) and belong to four major functional categories, namely, sensory neurons (perceiving specific inputs, such as mechanical stimuli), motor neuron (synapse with muscle cells), interneurons (receive signals from one neuron and sends to other neurons; largest group of neurons); and polymodal neurons (perform more than one of the above functions). Neuron subtypes covered in this review include six touch receptor cells (PVM, AVM, a pair of PLMs and a pair of ALMs), the GABAergic D-type motor neurons and ventral nerve cord, the RME motor neurons, the thermo-sensory AFD neuron, the ALA interneuron, the ASH sensory neuron, command interneurons and the oxygen-sensing URX neurons.

### 1.2 The *Caenorhabditis elegans* Canonical Apoptotic Cell Death Pathway

**FIGURE 1:**
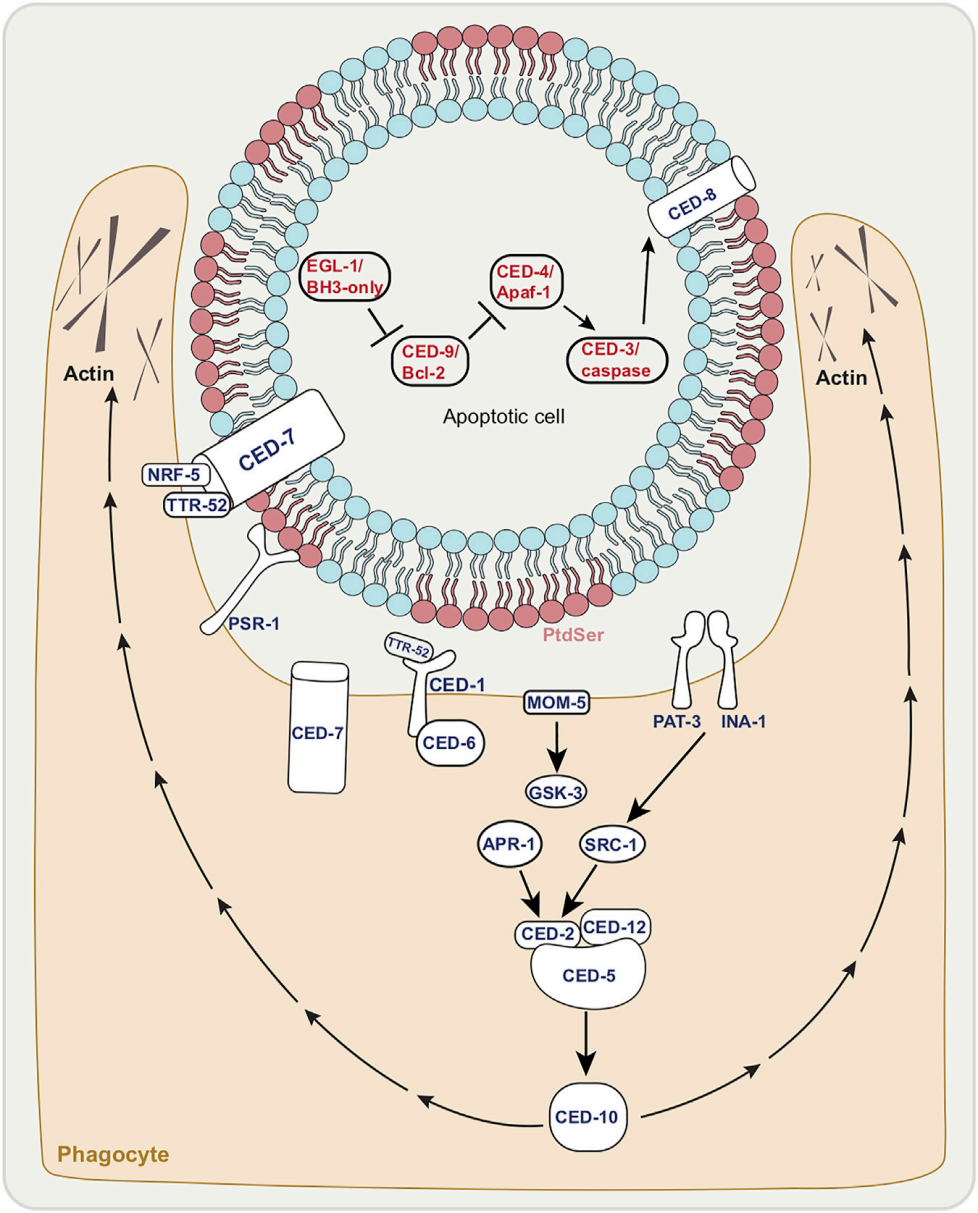
The canonical apoptotic cell death and engulfment pathways. Death execution by canonical apoptosis pathway factors (red letters); EGL-1/BH3-only activation leading to inhibition of CED-9/Bcl-2 allowing for CED-3/caspase activation by CED-4/Apaf-1. CED-8/Xk facilitates phosphatidyl serine (PtdSer) externalization of dying cell. Corpse with exposed PtdSer enables the recognition by phagocyte shown in tan. Activation of engulfment pathways factors (dark blue letters), allows for cytoskeletal rearrangement of phagocyte (extended pseudopods).

Programmed cell death is a critical event in metazoan development (Ghose and Shaham 2020). Apoptosis is a type of programmed cell death characterized by distinct ultrastructural hallmarks including condensed chromatin, compaction of the cytoplasm and membrane blebbing, with intracellular organelles remaining intact well into death. Apoptosis is seen during *C. elegans* development: in hermaphrodites, 131 of 1090 somatic cells originally generated die apoptotically, as do half of germ-line cells. That apoptotic death takes place through a defined program, controlled by specific genes first found in *C. elegans* (Figure 1). The main components of the *C. elegans* apoptotic pathway (Figure 1 **)** are conserved across different animal species and several excellent reviews cover this topic in depth (Fuchs and Steller 2011; Conradt et al., 2016; Wang and Yang 2016).

The most downstream core component of the apoptotic pathway is the caspase CED-3—with *C. elegans* studies first showing that caspases in general play a role in apoptosis (Horvitz 2003). Caspases (cysteine-aspartic acid proteases) are a group of aspartate-directed cysteine proteases. They are activated following cleavage of an inactive precursor at specific aspartates (Thornberry et al., 1997). In *C. elegans*, the main caspase CED-3 is the core conserved apoptotic cell death executioner (Yuan et al., 1993). There are three additional caspases, CSP-1, -2, and -3, (Shaham 1998). Of these, CSP-1 may have pro-apoptotic functions, whereas CSP-2 and CSP-3 appear to prevent CED-3/caspase auto-activation. Unlike loss of CED-3/caspase, in which case complete loss of function prevents apoptotic death, mutations in these three caspases only weakly impact apoptosis (Geng et al., 2009). We speculate here that these other caspases may have non-cell death functions yet to be discovered. For example, CSP-1 promotes the death of a subset of cells that are destined to die during *C. elegans* embryogenesis (Denning et al., 2013). As *csp-1* is expressed early on, it is conceivable that this gene may take part in aspects of neuronal development in non-cell death roles.

There are three main upstream regulators of the CED-3/caspase, also conserved. Immediately upstream of CED-3/caspase is the adaptor protein and CED-3/caspase activator CED-4/Apaf-1, which promotes CED-3/caspase function through cleavage of the inactive CED-3/caspase precursor. CED-4/Apaf-1 is a part of the apoptosome, a complex thought to bring caspase precursors into close proximity (Zou et al., 1999) to allow for cross activation. In *C. elegans,* the apoptosome is made up of eight CED-4/Apaf-1 adapter moieties which bind to two CED-3/caspase molecules (Qi et al., 2010). Across phyla, the apoptosome is in turn regulated by proteins of the BCL-2 family which reside on the mitochondrial outer membrane. In *C. elegans,* the BCL-2 related protein CED-9/Bcl-2 normally binds CED-4/Apaf-1 thus preventing apoptosome activation (Chinnaiyan et al., 1997; Spector et al., 1997; Wu et al., 1997) and hence CED-3/caspase function.

CED-9/Bcl-2 activity is controlled and negatively regulated by the binding of the BH3-only (BCL-2 homology domain 3) protein EGL-1/BH3-only. The *egl-1* gene is in turn regulated by transcriptional activation (Nehme and Conradt 2008; Nehme et al., 2010; Jiang and Wu 2014). CES-1, a member of the Snail family of transcription factors, regulates cell death (Ellis and Horvitz 1991; Yan et al., 2013) in specific lineages (the NSM neurosecretory motor neuron and the I2 interneuron) as well as asymmetric cell division and cell proliferation (Hatzold and Conradt 2008; Wei et al., 2017; Wei et al., 2020). CEP-1, the homolog of the mammalian p53 tumor suppressor, is required for apoptosis in the germline following DNA damage (Schumacher et al., 2005). Both regulate *egl-1*/*BH-3-only* transcription (Thellmann et al., 2003; Derry et al., 2007).

What does this core apoptotic pathway converge on? What are the CED-3/caspase cleavage targets? While the optimal substrate peptide sequence for CED-3 is thought to be DEXD, similar to that of mammalian caspase-3 and -7 (Thornberry et al., 1997), bona fide *in vivo* caspase substrates have been difficult to identify. One example in both worm and mouse is a member of the Xk-family of proteins, CED-8 in worms, which regulate plasma-membrane lipid asymmetry (Stanfield and Horvitz 2000; Suzuki et al., 2013). Normal cell plasma membranes show phospholipid asymmetry (Figure 1). The phospholipid phosphatidylserine (PtdSer) is normally confined to the inner leaflet, but is externalized to the outer leaflet of apoptotic cells following cleavage of CED-8/Xk, which allows for these cells to be recognized by phagocytes (Fadok et al., 1992), with PtdSer acting classically as an “eat me” signal (Figure 1).

### 1.3 The *Caenorhabditis elegans* Core Apoptotic Engulfment Machinery

Apoptotic cell death in *C. elegans* is tightly linked with apoptotic corpse engulfment. Phagocytosis (Ghose and Wehman 2021) is a vital process by which cellular debris are removed. In the context of cell death, phagocytosis involves a series of steps: a phagocytic cell recognizes the corpse that presents PtdSer, extends its plasma membrane to engulf the corpse and contain it within a limiting membrane (a phagosome) culminating in the intracellular degradation of the corpse via phagolysosomes. *C. elegans* lacks professional phagocytes. Thus, corpses are removed by neighboring cells such as hypodermal, body wall muscle, and gonadal sheath cells.

Here we mention the core members of the apoptotic engulfment machinery, which are conserved (Figure 1). This topic too has been reviewed extensively (Conradt et al., 2016; Wang and Yang 2016; Ghose and Wehman 2021). The two main pathways that regulate recognition of dying cells are parallel and partially-redundant (Ellis and Horvitz 1991). One pathway includes the membrane receptor CED-1/MEGF10 which is thought to recognize the PtdSer on the surface of dying cells (Zhou et al., 2001). CED-6/GULP is an adapter protein in this pathway that may play a role in signal transduction (Liu and Hengartner 1998; Liu and Hengartner 1999). CED-7 is a membrane ATP-binding cassette (ABC) transporter that may be involved in PtdSer presentation (Wu and Horvitz 1998). While CED-1/MEGF10 and CED-6/GULP function in the engulfing cell, CED-7/ABC transporter is required in both dying and engulfing cells (Wu and Horvitz 1998).

The second main pathway comprises the proteins CED-2/CrkII, CED-5/DOCK180, CED-10/Rac GTPase, and CED-12/ELMO (Reddien and Horvitz 2000; Gumienny et al., 2001; Wu et al., 2001; Zhou et al., 2001), which are thought to function together as a guanine nucleotide exchange factor (GEF) signaling module to control CED-10/Rac GTPase activity. CED-10/Rac GTPase is thought to control actin cytoskeletal assembly during the extension of the engulfing cell pseudopods around the dying cell. CED-5/DOCK180 and CED-12/ELMO have been shown to act downstream of at least 3 receptors involved in cell corpse recognition. These include PSR-1 (Reddien and Horvitz 2000; Wang et al., 2003), the Frizzled homolog MOM-5, and the integrin heterodimer INA-1/PAT-3 (Wang et al., 2003; Cabello et al., 2010; Hsu and Wu 2010). A partially redundant engulfment pathway to PSR-1 consists of the secreted PtdSer-binding protein TTR-52/transthyretin (Wang et al., 2010) the lipid-binding protein NRF-5 (Zhang et al., 2012), the membrane-bound CED-7/ABC transporter (Wu and Horvitz 1998), the transmembrane receptor CED-1/MEGF10 (Zhou et al., 2001), and the intracellular adaptor CED-6/GULP (Liu and Hengartner 1998).

A number of genes have also been identified for stages of phagocytosis that follow recognition and engulfment. Several genes have been implicated in phagosome maturation (Ghose and Wehman 2021). This entails formation of a phagosome vesicle after closure or sealing of the phagocyte pseudopods around the corpse. This is followed by the transition of this phagosome containing the corpse from an early to a late stage with increasing acidification by fusion with lysosomes and the final digestion of the corpse. Recently the cell-cell fusion protein EFF-1 has been shown to be involved in the phagosome sealing step (Ghose et al., 2018).

Here we focus primarily on genes required for the recognition and engulfment steps of apoptotic corpse clearance which have been shown to have non-canonical functions in neurons. It will be interesting to consider non-canonical roles of genes involved in phagosome maturation as well. For example, could there be a relationship or commonalities in the molecular mechanism with synaptic vesicle release or extracellular vesicle release from neurons, given that these events all involve some level of membrane fusion?

## 2 Non-Canonical Roles of Apoptotic Genes in the Nematode Nervous System

Non-cell death functions of apoptotic genes in the mammalian nervous system have been discussed thoroughly in some insightful reviews (Unsain and Barker 2015; Hollville and Deshmukh 2018). In the past 10 years, a number of non-cell death roles of *C. elegans* apoptotic genes have been described in the nematode nervous system. These include events at the origin or birth of neurons, aspects of neuronal function, response to injury and developmental pruning. We discuss these studies below.

### 2.1 Asymmetric Cell Division of Neuroblasts

Non-apoptotic functions of cell death genes can be observed in neuronal precursor cells. CED-3/caspase and its upstream regulators have been shown to play an active role in the asymmetric cell divisions of neuroblast mothers by controlling both size (generating one large and one small daughter) and fate (larger daughter lives, smaller daughter dies) (Chakraborty et al., 2015; Mishra et al., 2018). Undifferentiated neuroblasts have two possible fates: either to die (more “apoptotic potential”) or to further divide (more “mitotic potential”) to form neurons and the two types of potentialities antagonize each other such that one daughter has more of one potential. This has been reported in the NSM (neurosecretory motorneuron) neuroblasts (NSMnb) and the QL.p neuroblast.

**FIGURE 2:**
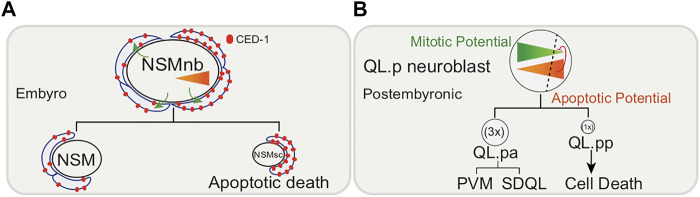
Embryonic and post-embryonic asymmetric cell division of neuronal precursors. **(A)** Size and fate of embryonic NSM (neurosecretory motorneuron) neuroblasts (NSMnb) controlled by CED-1/MEGF10-depenedent CED-3/caspase gradient. Larger daughter cell differentiates into NSM neuron while smaller cell, NSMsb, undergoes apoptosis. **(B)** QL.p neuroblast postembryonic asymmetric cell division controlled by PIG-1/MELK dependent gradient of mitotic potential antagonized by CED-3/caspase. Larger daughter cell, QL.pa differentiates to form PVM and SDQL neurons, while smaller daughter cell, QL.pp undergoes apoptosis. Triangles indicate gradient of apoptotic (orange) and mitotic (green) potential.

The two bilaterally symmetric embryonic NSMnbs divide asymmetrically each giving rise to a small cell (which dies), the NSM sister cell (NSMsc), and a large cell, the NSM (which survives and differentiates into a serotonergic motor neuron (Sulston et al., 1983; White et al., 1986; Chakraborty et al., 2015) (Figure 2A). The embryonic NSM neuroblast lineage also shows a gradient of CED-3/caspase activity in the mother of cells fated to die. The phagocytic receptor CED-1/MEGF10 is necessary for the gradient of CED-3/caspase activity in the NSM neuroblast, and the nonrandom segregation of active CED-3/caspase into the smaller NSMsc, where it promotes apoptotic cell death (Chakraborty et al., 2015; Lambie and Conradt 2016).

This trend is also observed post-embryonically. During the first larval stage of postembryonic development, the QL.p neuroblast divides asymmetrically to produce a larger anterior daughter, QL.pa, which survives and divides further to form the PVM and SDQL neurons and a smaller posterior daughter, QL. pp, which is fated to die (Sulston and Horvitz 1977; Cordes et al., 2006) (Figure 2B). Daughter cell size and fate are thought to be functionally coupled, given that defects in the asymmetric division of QL.p by size can affect the fate of its daughters (Cordes et al., 2006; Singhvi et al., 2011; Gurling et al., 2014; Teuliere et al., 2014; Teuliere and Garriga 2017). A *pig-1/MELK* (maternal embryonic leucine zipper kinase)-dependent gradient of “mitotic potential” is formed in the QL.p neuroblast, and CED-3/caspase and the core apoptotic pathway are thought to antagonize this “mitotic potential”. PVM and SDQL are formed regardless, but if what would have been the smaller cell does not die, its fate changes to becoming PVM, SDQL or to remain undifferentiated. While the engulfment gene *ced-1/MEGF10* is required for apoptosis, it is not required for the asymmetric cell division by size.

Caspase gradients are also observed in mammals/flies in terminally differentiated neurons and are important for region-specific regression or pruning during development (Simon et al., 2012; Maor-Nof and Yaron 2013). Whether nematode neurons undergoing pruning or death show such gradients remains to be determined.

### 2.2 Neuronal Function and the Pro-Apoptotic EGL-1/BH3-Only

Once a neuron is formed, it becomes part of a circuit. *C. elegans* exhibits various complex behaviors governed by the intricate architecture of neuronal circuits of its relatively simple nervous system. Behaviors include foraging, mating (Liu and Sternberg 1995), sleep (Raizen et al., 2008), and avoidance response (Bargmann et al., 1993; Zhang et al., 2005). As discussed below, cell death genes that regulate *egl-1/BH3-only* transcription can mediate behavior independent of cell death. Additionally, *egl-1/BH3-only* can be actively transcribed in neurons without inducing cell death, suggesting new functions.

#### 2.2.1 The Scratch Family Transcriptional Repressor Family Homolog CES-1 Regulates Glutamatergic Behavior

**FIGURE 3:**
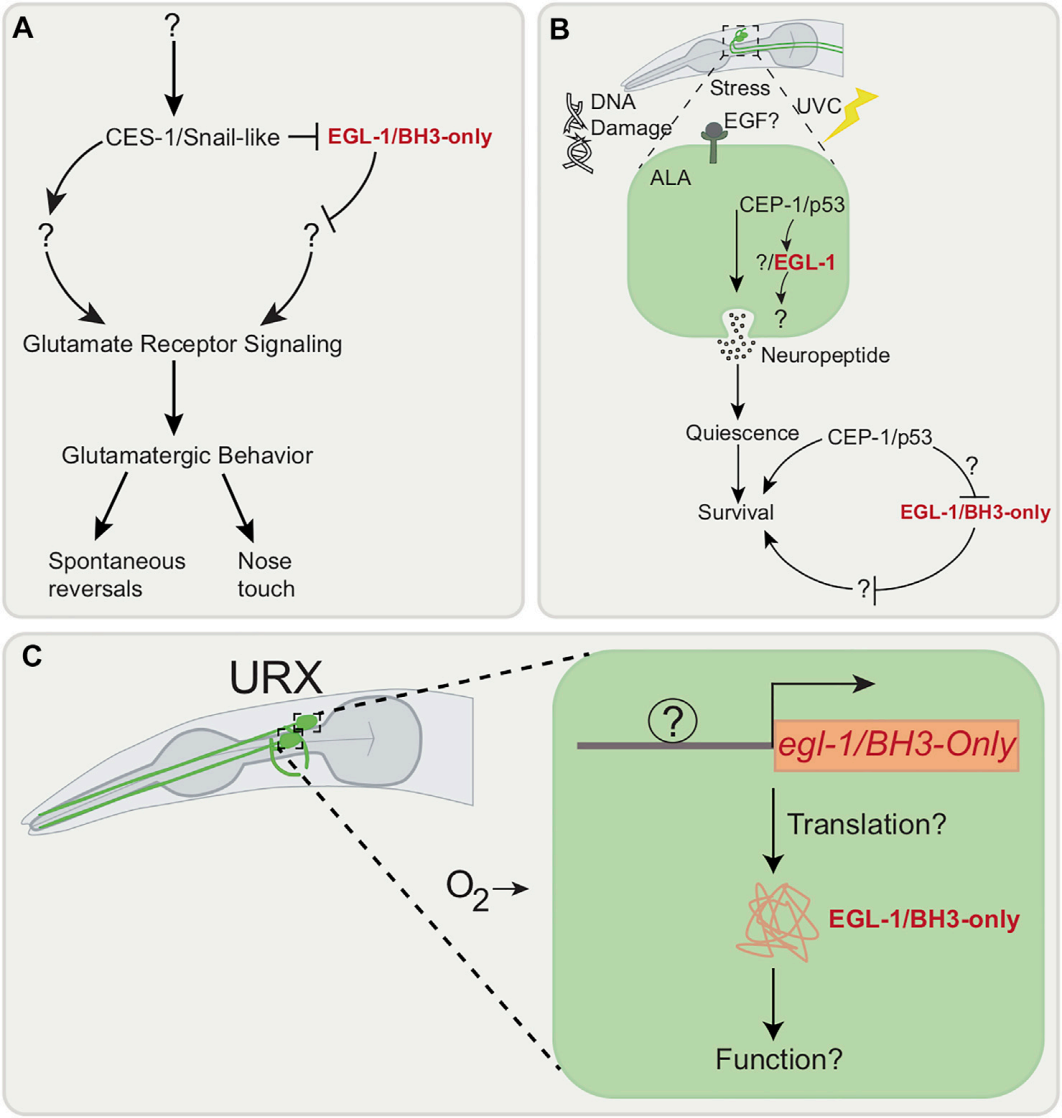
Cell death genes associated with neuronal outputs. **(A)** Model suggesting that glutamatergic behavior (spontaneous reversal and nose touch response) is controlled by CES-1/Snail-like. CES-1/Snail-like appears to function in a partially cell autonomous manner in GLR-1 expressing neurons and its regulators or targets are unknown. **(B)** EGF stress-induced signaling following Ultraviolet C (UVC) irritation in the ALA neuron activates CEP-1/p53 which promotes transcription of EGL-1/BH3-only. Location of where CEP-1/p53 is functioning in ALA neuron remains unknown. **(C)** *egl-1/BH3-only* is transcribed post-embryonically in URX neuron without inducing apoptosis. Whether the EGL-1/BH3-only is translated in URX neuron and its exact function remains unknown.

*C. elegans* glutamatergic behavior, which includes gentle nose touch and spontaneous reversals, is attributed to mechanosensory neurons located in the nematode head, including ASH, FLP, QLQ (Kaplan and Horvitz 1993). Stimulation of the ASH neuron activates command interneurons and the consequent behavioral outputs require the AMPA-type glutamate receptor (AMPAR) subunit GLR-1 (Maricq et al., 1995). A behavioral RNAi screen implicates the cell death gene, *ces-1*, which encodes the mammalian scratch family transcriptional repressor family homolog, in the regulation of glutamatergic behavior (Park et al., 2021) (Figure 3A). As mentioned, CES-1 is a cell death regulator for the NSM neuron (Ellis and Horvitz 1991) and the I2 interneurons (Peden et al., 2008). In these cells, CES-1 represses the pro-apoptotic *egl-1* (Ellis and Horvitz 1991). It is also involved in asymmetric cell division of the NSM sister cells and in cell proliferation (Yan et al., 2013). CES-1 has been suggested to bidirectionally regulate glutamatergic signaling. Defects in glutamatergic behavior due to loss of *ces-1* appear not to be due to death of ASH neurons, defects in muscle function or neuromuscular junctions (NMJ). Additionally, *ces-1* appears to act partially in a cell non-autonomous manner. Interestingly, while *ces-1* appears to be important for glutamatergic behavior in young adults, *ces-1* expression is not upregulated at this stage (Park et al., 2021), suggesting that CES-1 may affect the transcription of a factor earlier on in development. The direct transcriptional target of CES-1 in GLR-1*-*expressing neurons remains to be identified. One possibility is that *egl-1/BH3*-only transcriptional regulation by CES-1 is important for glutamatergic behavior. As mentioned below, *egl-1/BH3*-only may be transcribed without causing cell death.

#### 2.2.2 CEP-1, the Homolog of Mammalian p53, Promotes Stress-Induced Sleep

Quiescence behavior, defined as the absence of movement, is a “sleep-like” state observed and described across various species (Siegel 2009). In *C. elegans*, this behavior can be observed in two instances: during the molting stages and in the presence of stress. This sleep-like state in the nematode is characterized by a significant reduction in response to stimuli, body movement and pharyngeal pumping (Raizen et al., 2008; Nagy et al., 2014). Quiescence observed when the nematode is exposed to stressors is termed Stress-Induced Quiescence (SIQ) or Stress Induced Sleep (SIS) (Hill et al., 2014; Trojanowski et al., 2015). SIS is dependent on the ALA neuron (Trojanowski et al., 2015).

SIS begins with exposure to cellular stress, which in turn prompts cells to release epidermal growth factor (EGF) signals (Van Buskirk and Sternberg 2007). The EGF signals then causes the release of neuropeptides by the ALA neuron leading to SIS (Van Buskirk and Sternberg 2007). Through a candidate gene approach, *cep-1,* which encodes the mammalian p53 tumor suppressor homolog in nematodes, which is also a DNA damage response gene in the germline (Schumacher et al., 2001), has been found to be involved in SIS induction after Ultraviolet C (UVC) irradiation (DeBardeleben et al., 2017) (Figure 3B).

The nematode cellular stress response and repair pathway is similar to that of the mammalian one when the animal is exposed to UV radiation (Jolliffe and Derry 2013). When DNA damage is detected, CEP-1/p53 induces apoptosis by increasing transcription of pro-apoptotic *egl-1/BH3-only* and *ced-13* in germline cells (Jolliffe and Derry 2013). Two genes are known to be involved in DNA damage response pathway, namely, *cep-1/p53* and its regulator *atl-1/ATR* (Jolliffe and Derry 2013). The recovery response after UV exposure in the nematode includes altered pharyngeal pumping, body-movement, and olfactory avoidance (DeBardeleben et al., 2017). The general assumption is that when cells experience stress-induced damage, the organism enters a state of sleep. In this state, resources that would be necessary for behavior are then re-allocated to repair cellular functions (DeBardeleben et al., 2017). Interestingly, *atl-1/ATR* (Jolliffe and Derry 2013) is not involved in these behaviors, while CEP-1/p53 is involved in UVC-induced movement quiescence after UVC irradiation: loss of *cep-1/p53* suppresses EGF-mediated body movement quiescence. This suggests that *cep-1/p53* is regulated by genes other than *atl-1/ATR.* The exact location of where CEP-1 functions remains to be found, but it is speculated to be downstream or parallel to EGF activation of the ALA (DeBardeleben et al., 2017).

#### 2.2.3 The Pro-apoptotic *Egl-1/BH3-Only* can Be Transcribed in Response to Neuronal Activity Without Inducing Cell Death

Transcription of *egl-1/BH-3-only* in response to activity has been shown to occur post-embryonically without inducing apoptotic cell death in the adult URX neuron pair along with AQR, PQR and one of the AWXon/off neuron pair (Cohn et al., 2019) (Figure 3C). Located in the head of the nematode, the URX neurons are mainly involved in oxygen sensing (Zimmer et al., 2009) and immunity (Zhang and Zhang 2009). In URX, *egl-1/BH3-only* transcription requires sensory transduction: loss of guanylyl-cyclases, necessary for URX to respond to changes in ambient oxygen (Cheung et al., 2004), result in loss of *egl-1/BH3-only* transcription. While *egl-1/BH3-only* transcription is evident, it is not known whether the protein is translated and the role of this gene in the URX is unknown as loss of *egl-1*/BH3-only does not cause any impairment in URX-related phenotypes. It is possible that the role of EGL-1/BH3-only is only evident under very specific conditions, such as under a preconditioning paradigm. In other systems several BH3-only members are involved in non-apoptotic functions that include DNA repair, cell cycle regulation and metabolism (Lomonosova and Chinnadurai 2008). It will be exciting to discover novel, non-apoptotic targets of EGL-1/BH3-only in neurons. Additionally, other neurons that may show *egl-1/BH3-only* transcription such as glutamatergic neurons, which may have been missed owing to missing promoter regulatory elements in the reporter used in this study. This can be overcome through a transcriptomics approach. It is also possible that the URX has an as yet unassigned function that depends on *egl-1/BH3-only*.

### 2.3 Axon Regeneration Following Injury

Most studies over the past decade on non-canonical apoptotic gene roles in *C. elegans* neurons focus on regeneration, a conserved mechanism of neuronal repair following injury. *C. elegans* neurons have a natural ability to repair following laser severing (such as femtosecond laser axotomy) without damage to the nearby tissue (Yanik et al., 2004; Hammarlund et al., 2009). This can be visualized *in vivo* by labelling neurites with GFP. There has been much interest in deciphering the molecular mechanism behind axon regeneration/neuronal reconnection with work largely focused on axons, though it will be interesting to extend studies to dendrites.

**FIGURE 4:**
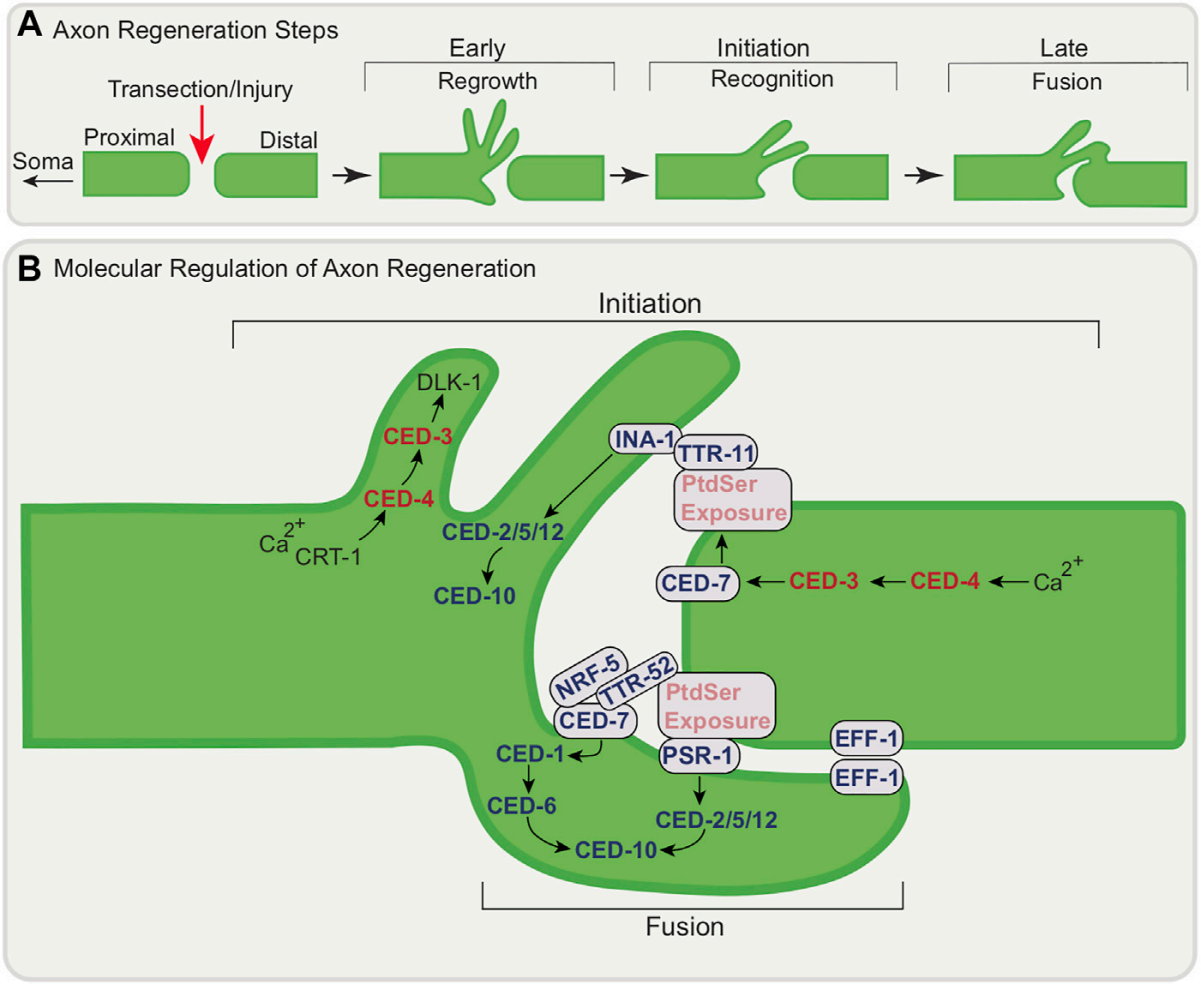
Conceptual model of the molecular regulation of axon regeneration at the transection site by apoptotic genes. **(A)** Schematic of axon regeneration steps following injury; regrowth, recognition, and fusion **(B)** Axon Regeneration Initiation: CRT-1-dependent calcium release following injury leads to local activation of CED-4/Apaf-1 and subsequent activation of CED-3/caspase and DLK-1 to initiate filopodia extension. CED-7/ABC transporter function is also promoted by CED-3/caspase leading to PtdSer exposure. INA-1/PAT-3 may recognize PtdSer via TTR-11 leading to the activation of the CED-2/CrkII-CED-5/Dock180/CED-12 Elmo signaling module which in turn activates CED-10/Rac GTPase. Axon Regeneration-Fusion: PSR-1 functions cell autonomously in regenerating neuron through a pathway involving TTR-52/transthyretin, NRF-5, CED-7/ABC transporter and CED-6/GULP to promote axonal fusion, a late step of regeneration. Fusion also involves EFF-1 accumulation at the cut site of the axon mediating fusion of distal and proximal process. For simplicity, all regeneration steps are shown at one proximal-distal segment junction following injury.

Axon regeneration following nervous system injury takes place through defined steps (Figure 4A) through which transected soma-distal axons reconnect with their original soma-proximal segment. The soma-proximal axon segment, which is still attached to the soma/cell body, regrows towards and forms filopodia and growth cones reconnects, and fuses with the severed distal fragment. In *C. elegans* neurons, there are well characterized axonal injury models for the ALM and PLM mechanosensory neurons as well as the GABAergic D-type motor neurons. ALM mechanosensory neurons and D-type motor neurons normally mount an efficient early response (with a reconnection rate of more than 50%) to injury allowing for rapid regeneration of severed axons. After axotomy in adults, the severed processes persist and remain functional. Several studies (Yanik et al., 2004; Wu et al., 2007; Bourgeois and Ben-Yakar 2008; Gabel et al., 2008; Hammarlund et al., 2009) have reported that the severed process regenerates from the soma-proximal side by initially forming short filopodia with further axonal extensions. Interestingly, the distal segment also shows regenerative outgrowth tendencies. From these paradigms we know that the c-Jun N-terminal kinase (JNK) MAP kinase (MAPK) pathway, or JNK–MAPK pathway, acts as a major regulator of axon regeneration initiation and may also play a role in the sensing of axonal damage (Nix et al., 2011; Li et al., 2012; Li et al., 2015).

#### 2.3.1 CED-3/Caspase and CED-4/Apaf-1 Act in the Early Steps of Axon Regeneration

Cell death genes have been shown to be part of a conserved pathway for neuronal reconstruction and remodeling (Pinan-Lucarre et al., 2012). CED-3/caspase acts in the early regenerative response: in its absence the initial outgrowth (both rate and extent) response is impaired and the re-association of the severed axon segments is delayed. CED-3/caspase activity is necessary and *ced-3/caspase* acts in the damaged neuron (Figure 4B). CED-4/Apaf-1 also promotes regeneration with a subdomain of the caspase recruitment domain (CARD) of CED-4/Apaf-1 regulating CED-3/caspase (Wang et al., 2019). While CED-3/caspase and CED-4/Apaf-1 are involved in initial outgrowth and rapid reconnection, they are not essential for growth per se: loss of these genes does not affect regeneration long term. As discussed below, other genetic pathways are involved to promote later steps of regeneration. While facilitating rapid reconnection at early stages is an important function for these cell death genes, upstream members of the core apoptotic pathway EGL-1/BH3-only and CED-9/Bcl-2 are not required. Calcium signaling is a feature of neuronal responses to damage and the ER calcium-storing chaperone CRT-1/Calreticulin appears to act upstream of CED-3/caspase in the same pathway that promotes initial outgrowth. CED-3/caspase appears to act upstream of DLK-1, a conserved kinase linked to regeneration across species to promote axon regeneration (Nix et al., 2011).

#### 2.3.2 Engulfment Machinery in Regeneration Initiation

The initiation of axon regeneration of D-type motor neurons (which extend their axons dorso-ventrally) following laser surgery has been shown to involve apoptotic engulfment genes (Pastuhov et al., 2016). Following axon injury, PtdSer accumulates around the injured axons as an injury and “save me” signal (Neumann et al., 2015) (Figure 4B). For the D-type motor neurons, this depends on TTR-11/transthyretin (TTR)-like secreted protein, together with CED-7/ABC transporter as well as CED-3/caspase. As suggested above, CED-3/caspase is activated by intracellular calcium signaling involving CRT-1/Calreticulin as a response to injury, likely activating CED-4/Apaf-1 only locally. CED-3/caspase likely cleaves the C-terminal region of CED-7/ABC transporter. CED-7/ABC transporter facilitates the translocation of PtdSer to the outer leaflet of the plasma membrane of the injured axon. INA-1/PAT-3 may recognize exposed PtdSer through the bridging molecule, TTR-11 (Hisamoto et al., 2018). TTR-11/transthyretin binds to both the extracellular domain of INA-1/integrin and PtdSer. Interestingly, TTR-11/transthyretin can act cell non-autonomously and is in fact not expressed in the regenerating D-type motor neurons. INA-1/integrin signals through the CED-2/CrkII—CED-5/DOCK180– CED-12/ELMO signaling module. This in turn activates CED-10/Rac GTPase. CED-10/Rac GTPase acts as the upstream regulator of MAX-2, a Ste20-related protein kinase, ultimately activating MLK-1 MAPKKK, the most upstream component of the core JNK pathway and the activation of which is important for signal specificity. This culminates in axon regeneration.

#### 2.3.3 Role of Apoptotic Engulfment Genes in Later Stages of Regeneration Through Axonal Fusion

Apoptotic engulfment genes have also been shown to be involved later in regeneration following axotomy. Axonal fusion, which occurs in many invertebrate species, is a spontaneous event that re-establishes the connection between the soma-attached proximal part of the axon and the separated distal fragment (Hoy et al., 1967; Birse and Bittner 1976; Deriemer et al., 1983; Macagno et al., 1985; Bedi and Glanzman 2001). Axonal fusion, best-studied in *C. elegans* in the PLM mechanosensory neurons (Ghosh-Roy et al., 2010; Neumann et al., 2011) following axotomy, allows for the full recovery of neuron function (Abay et al., 2017).

In addition to apoptotic cell engulfment, PtdSer and PSR-1 also play important roles in axonal fusion (Figure 4B) (Neumann et al., 2015). PtdSer is exposed on the injured axon (Neumann et al., 2015). PSR-1 functions in axonal fusion differently from its canonical role in engulfment signaling. First, it functions in axonal fusion cell-autonomously, in the regenerating neuron. Second, it functions in a pathway parallel to its canonical pathway that involves the transthyretin protein TTR-52/transthyretin which binds to PtdSer to promote fusion following injury. This pathway also involves CED-7/ABC transporter, NRF-5 and CED-6/GULP (Liu and Hengartner 1998; Wu and Horvitz 1998; Wang et al., 2003; Zhang et al., 2012). In this model, PtdSer serves as a ‘save-me’ signal for the distal fragment to re-establish axonal integrity. Further studies have shown that the ability of PLM to undergo axonal fusion is strongly influenced by the amount of PtdSer exposed after injury (Abay et al., 2017).

The nematode-specific fusogen, epithelial fusion failure 1 (EFF-1), a trimeric cell-cell fusion protein similar to class II viral fusion proteins, has been shown to be involved in axonal fusion (Ghosh-Roy et al., 2010; Neumann et al., 2011). EFF-1 has also recently been shown to promote phagosome sealing (Ghose et al., 2018). The mechanism behind EFF-1-mediated axonal fusion during regeneration of the PLM neuron has been described (Neumann et al., 2015) (Figure 4B). EFF-1, which is normally broadly distributed in the PLM cell body and axon, undergoes dynamic localization changes following axonal injury. Soon after axotomy, EFF-1 accumulates at the axonal membrane at the cut site-at the tips of both the proximal and distal segments. Following regeneration, EFF-1 localizes to the growth cone membrane, perhaps to mediate fusion once contact is regained.

### 2.4 Non-Apoptotic Cell Death

Necrosis (Crook et al., 2013) is another form of cell death with morphological features differing from apoptosis that include excessive cell swelling, membrane disruption and blebbing, nuclear disruption and DNA fragmentation (Syntichaki and Tavernarakis 2002; Golstein and Kroemer 2007; McCall 2010). Cells respond to necrosis-inducing insults through genetically controlled death programs. The core apoptotic killing genes are not required for death in established models of necrosis in *C. elegans,* though the apoptotic engulfment genes are required (Chung et al., 2000). Among neurons, necrosis models involve gain-of-function mutations in “degenerin” ion channel subunits. Dominant, gain-of-function mutations in the degenerin gene mec-4, for example, result in a hyperactive ion channel and cause the six touch receptor cells (PVM, AVM, a pair of PLMs, and a pair of ALMs) expressing it to necrose (Driscoll and Chalfie 1991).

#### 2.4.1 CED-4/Apaf-1 in Neuronal Necrosis

In *C. elegans*, necrosis and apoptosis are believed to function as separate pathways (Gumienny et al., 2001). However, depletion of coenzyme Q (CoQ), which is a necessary component of the mitochondrial electron transport chain, in GABA motor neurons in the ventral nerve cord results in a cell death mechanism that includes aspects of both apoptotic and necrotic pathways (Earls et al., 2010). Other neuron classes do not respond as dramatically. Phenotypically, GABA neurons cell bodies swell, consistent with a necrotic mechanism. The appearance of degenerating GABA neurons correlates with the occurrence of an uncoordinated (Unc) behavior phenotype. A subset of the core apoptotic genes, *ced-4/Apaf-1* and *ced-3/caspase*, are required for degeneration: mutations in both suppress degeneration of both the ventral nerve cord processes and soma. CoQ reduction in the mitochondria appears to signal CRT-1/Calreticulin-dependent Ca^2+^ release from the endoplasmic reticulum. Ca^2+^ may activate CED-4/Apaf-1, as suggested earlier here. In addition to specific components of the apoptotic pathway, the dynamin-related protein DRP-1 that promotes mitochondrial fission is also required for degeneration. Surprisingly, while mutations in *ced-4/Apaf1* strongly suppress the uncoordinated phenotype, mutations in *ced-3/caspase* do not. This suggests CED-4/Apaf-1 is regulating another factor that acts in parallel to CED-3/caspase. Could this be one of the other caspases? This begs the question of what new molecules exist that act downstream of CED-4/Apaf1. Another question is why such a specific subset of neurons are affected by CoQ depletion. This highlights that cell death genes function differently in different neuron types.

### 2.5 Localized Elimination

The function of the core apoptotic cell death genes has traditionally been associated with destruction of the entire cell. This typically only considers cells with simple architecture. However, neurons, as many other cell types, have more intricate structures. This directs us to several questions. Do the core cell death pathway genes function in their canonical fashion in different parts of the cell, namely in the extensions or neurites? Would a caspase have different substrates in different parts of the cell? Do engulfment genes treat different types of cell debris differently?

Given the complexity of neuron structure, neuronal elimination can take place in a localized manner, without loss of the entire neuron, where, for example, only the axon degenerates. This can be a feature of neurodegenerative disease and neuronal injury. One form of region-specific elimination in the nervous system is developmental neurite pruning (Schuldiner and Yaron 2015; Ghose and Shaham 2020) which removes supernumerary connections to refine and sculpt. This has been demonstrated in flies and mammals (Technau and Heisenberg 1982; Bagri et al., 2003; Watts et al., 2003; Williams et al., 2006). One form of pruning entails the fragmentation of an axon or dendrite. Another form of developmental pruning is process retraction, or “dying back”, without fragmentation. Caspases are important for pruning (Williams et al., 2006; Schoenmann et al., 2010; Simon et al., 2012; Simon et al., 2016). Another example of localized elimination is pruning or micropruning of synapses (Neniskyte and Gross 2017; Wilton et al., 2019; Zhao and Zeng 2021) which is important for neurons to make precise and appropriate connections and which also occurs in diseased states.

#### 2.5.1 Apoptotic Cell Killing Genes in Synapse Elimination

**FIGURE 5:**
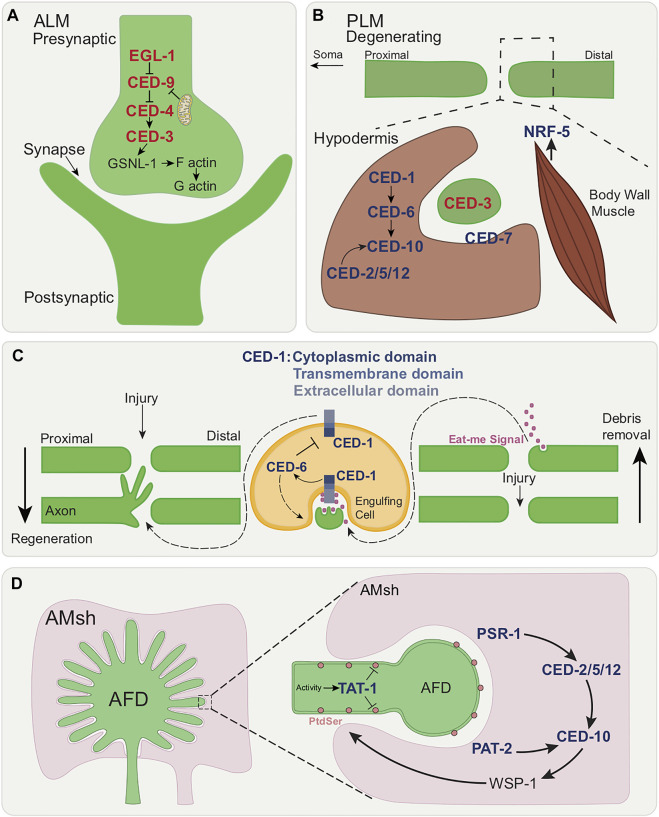
Roles of apoptotic cell death and engulfment genes in various forms of localized cell elimination. **(A)** Canonical apoptotic pathway functions cell autonomously in synaptic regions of the RME and DD motor neurons. Once activated by axonal mitochondria, canonical apoptotic pathway leads to the cleavage of GSNL-1, actin filament severing and culminating in synapse elimination. **(B)** Members of the canonical engulfment pathway function non-cell autonomously in the surrounding hypodermis to clear axonal debris following PLM axon degeneration. CED-1/MEGF10, CED-6/GULP and the CED-2/CrkII-CED-5/Dock180-CED-12/ELMO module activates CED-10/Rac GTPase. Additionally, CED-7/ABC transporter and NRF-5 function in the two-fold role of promoting and suppressing axon degeneration during development. **(C)** Dual functions of CED-1/MEGF10 within engulfing muscle cell in ALM axon regeneration and axon debris removal. Eat-me signal release following axon injury leads to recognition by CED-1/MEGF10. CED-1/MEGF10 extracellular/cytoplasmic domain located on the surface of engulfing muscle cell is required for regeneration. CED-6/GULP prevents axonal growth through the downregulation by CED-1 and promotes axon debris engulfment. **(D)** Model showing the removal of neuronal receptive endings of AFD neuron by AMsh glia. In a temperature-dependent manner, TAT-1/ATP8A inhibits PtdSer exposure and PSR-1 and PAT-2/a-integrin initiate selective engulfment by the activation of CED-2/CrkII-CED-5/DOCK180-CED-12/ELMO module and CED-10/Rac GTPase.

All four genes of the canonical apoptotic pathway have been shown to promote selective synapse elimination by facilitating the disassembly of the actin filament network (Meng et al., 2015) (Figure 5A). Synapse elimination of the GABAergic RME (dorsal and ventral) head motor neurons takes place during larval development. During the first larval (L1) stage, vesicles containing presynaptic components (including synaptobrevin and a presynaptic active zone protein) accumulate transiently at the dorsal/ventral neurite ends, getting pruned by the later L2 second larval stage. Unbiased genetic screens revealed a function of all four members of the core apoptotic pathway in synapse elimination in both the RME (dorsal and ventral) head motor neurons and the DD motor neurons. Loss-of-function mutations in all four core-apoptotic cell death genes show defects in localization of these pre-synaptic components, with a failure to eliminate these clusters. Furthermore, the apoptotic cell death pathway functions cell autonomously at synaptic regions and is activated by axonal mitochondria, whose presence is necessary for the synaptic localization of cell death proteins. The elimination of ventral synapses is suppressed in cell death mutants. But, unlike in RME neurons, the synapse elimination defects dramatically decreases with age suggesting other pathways may act in parallel with the cell death pathway in DD motor neurons (Meng et al., 2015).

The cell death pathway converges on the actin filament-severing protein, GSNL-1. GSNL-1 is the best characterized member of the actin-severing gelsolin-villin family in *C. elegans* and can sever actin filaments in a calcium-dependent manner. Filamentous actin (F-actin) is enriched at synapses and regulation of actin dynamics is important for neural development. The cell death pathway activates GSNL-1 through caspase-dependent cleavage. CED-3/caspase cleaves GSNL-1 at a conserved C-terminal target site. The cleaved active form of GSNL-1 severs actin filaments by actin depolymerization. F-actin accumulates in both RME and DD neurons in *gsnl-1* mutants suggesting the cell death pathway acts in a similar way in both neuron types. Activation of the cell death pathway is likely a universal mechanism for synapse elimination in *C. elegans*. Transient DD motor neuron synapses are eliminated in the ventral cord and new synaptic connections formed in the dorsal cord (White et al., 1978; White et al., 1986; Hallam and Jin 1998).

The study from Meng et al. is consistent with prior investigations. *In vitro* assays identified mammalian gelsolin as one of the first caspase substrates (Kothakota et al., 1997) and the cleavage of gelsolin by caspases has been shown to result in morphological changes of apoptotic cells (Kothakota et al., 1997). There are multiple points of novelty in Meng et al.: first, it unveils a conserved molecular mechanism of synapse elimination; second, it provides proof of an *in vivo* caspase target, GSNL-1; third, this study shows that GSNL-1 cleavage induces F-actin disassembly and is the first study showing a function of the gelsolin-villin family in *C. elegans* neurodevelopment; finally, it presents a new target of cell death machinery, that does not lead to cell death.

#### 2.5.2 Apoptotic Engulfment Genes in the Clearance of Axonal Debris Following Injury

In the case of axons, following injury or transection, Wallerian degeneration, described in various vertebrate species as well as Drosophila (Coleman and Freeman 2010), takes place and is marked by stereotypical degenerative steps. These include a latent period where the severed distal axon fragment persists followed by its thinning, beading, fragmentation, and eventual clearance by phagocytes. Wallerian degeneration slow (*Wld* ^S^) mutant mice show drastic delays in the onset of this degeneration (Lunn et al., 1989). Expression of the murine *Wld* ^*S*^ gene delays axonal degeneration in, rat, zebrafish, and Drosophila (Adalbert et al., 2005; Beirowski et al., 2008; Avery et al., 2009; Martin et al., 2010) suggesting this type of elimination is genetically regulated. While axonal degeneration is genetically distinct from apoptosis (Finn et al., 2000; Whitmore et al., 2003; Osterloh et al., 2012), the clearance of axonal debris shares molecular components with the clearance of apoptotic corpses (MacDonald et al., 2006; Ziegenfuss et al., 2012). Such clearance is important for neurons to recover from injury and to re-establish lost connections (Wahl et al., 1974; Vargas et al., 2010; Farah et al., 2011).

PLM axon degeneration following laser axotomy bears some morphological similarity to injury-induced Wallerian degeneration. However, the delay between the fragmentation and clearance steps is not observed and the process proceeds independently of the WLD^S^ and Nmnat pathway (Nichols et al., 2016). Interestingly there are developmental stage-dependent differences in the progression of degeneration, which occurs faster in the early larval stage. This is also true for GABAergic DD motor neurons, suggesting the developmental differences are a general feature of *C. elegans* neurons. Caspase function is not required for degeneration: mutants for *ced-3/caspase* and *ced-4/Apaf1*, genes of the canonical apoptotic pathway, show no axon degeneration defect. This is consistent with what is observed in vertebrates.

A non-cell autonomous role of members of the canonical engulfment pathway in the clearance of axonal debris has been uncovered in *C. elegans* (Nichols et al., 2016) (Figure 5B) This is reminiscent of what is known in other species in which clearance of axonal fragments by glial cells involves molecular components necessary in apoptotic engulfment. In the nematode, the transmembrane receptor CED-1/MEGF10, the adaptor protein CED-6/GULP, the CED-2/CrkII, CED-5/DOCK180, and CED-12/ELMO GEF module and CED-10/Rac GTPase play roles in axonal debris clearance (Nichols et al., 2016). Here, CED-1/MEGF10 and CED-6/GULP function non-cell autonomously in the surrounding epidermis/hypodermis (which engulfs the axonal fragments and is hence phagocytic). Of note, the involvement of these engulfment genes is restricted to the early larval stage when the rate of axonal elimination occurs very rapidly.

Additionally, the CED-7/ABC transporter and the lipid-binding protein NRF-5 play a role in axonal clearance. CED-7/ABC transporter promotes axon degeneration early in development of larva and suppresses it in later stages thereby playing a dual role in axon elimination. CED-7/ABC transporter in the apoptotic context also has two functions: producing lipid-containing vesicles that promote clearance early on and then later removing of lipids from the apoptotic cell. With this in mind, it is possible that earlier in development CED-7/ABC transporter promotes lipid secretion (where lipids are an “eat me” signal) from the axon and later lipid removal, thus promoting and repressing degeneration respectively. How CED-7/ABC transporter makes this switch in function remains to be determined.

As described above, the re-growing axon can reconnect to its separated distal fragment through axonal fusion (Wu et al., 2007; Ghosh-Roy et al., 2010; Neumann et al., 2011) Interestingly, the same genes involved in axonal fusion are also involved in axon degeneration. Thus, axotomy induces in a competition between forces of axonal repair (fusion) and degeneration (clearance via hypodermis) each involving cell death genes.

#### 2.5.3 CED-1/MEGF10 has Distinct Non-Cell Autonomous Roles in Axon Regeneration and the Removal of Axonal Debris

Work on the ALM touch neuron shows that following axotomy, proximal axonal debris are cleared away and the axon regenerates coincidentally. CED-1/MEGF10 acts in the engulfing muscle cells to promote both events via two biochemically separable functions (Chiu et al., 2018) (Figure 5C). Loss of *ced-1/MEGF10* causes defects in axon debris removal and *ced-1/MEGF10*-expressing muscle protrusions are found tending towards axonal debris. CED-1/MEGF10-mediated phagocytosis appears to be involved, based on phagocytic markers. CED-1/MEGF10 is also proposed to act as an adhesion molecule contributed by neighboring muscle. This is presumed to promote guidance, outgrowth and regeneration. CED-1/MEGF10 is required for the formation of growth cones from exploratory filopodia and loss of *ced-1/MEGF10* results in major defects in axon regeneration, as opposed to a mere delay. The regeneration functions of CED-1/MEGF10 require its extracellular/cytoplasmic domain from the surface of the engulfing muscle cell. Moreover, the p38 MAPK pathway is important for CED-1/MEGF10-mediated axon regeneration. Additionally, CED-5/DOCK180 can function both cell-autonomously and non-cell-autonomously to promote axon regeneration and CED-6/GULP inhibits axon regrowth through downregulating CED-1/MEGF10. However, CED-1/MEGF10’s function in engulfing muscle cells for axon regeneration does not involve CED-5/DOCK180 or CED-6/GULP.

#### 2.5.4 Active Pruning of Sensory Neurons by Glia *Via* Apoptotic Engulfment Genes

Across species, glia are known to engulf associated neuron endings (Schafer and Stevens 2013; Freeman 2015; Wilton et al., 2019). Engulfment genes have been shown to play a role in post-developmental neuronal remodeling and in temperature sensing (Raiders et al., 2021) (Figure 5D). The sensory endings of the single AFD thermo-sensory neuron in adult *C. elegans* are actively engulfed by the AMsh glia in a manner that is regulated by temperature (Raiders et al., 2021). This takes place through use of components of the canonical apoptotic phagocytosis machinery. TAT-1 is an ortholog of mammalian translocase ATP8A PtdSer flippase phospholipid transporter which is required for PtdSer sequestration to the plasma membrane inner leaflet (Andersen et al., 2016). Loss-of-function results in increased apoptotic cell corpse engulfment (Hong et al., 2004; Darland-Ransom et al., 2008), as well as AFD ending engulfment. Engulfment is initiated through the combined action of TAT-1/ATP8A in the AFD and glial PSR-1 and PAT-2/a-integrin. The CED-2/CrkII, CED-5/DOCK180, CED-12/ELMO GEF complex then activates glial CED-10/Rac GTPase which promotes the activation of actin remodeling WSP-1/nWASPp. Engulfment-defective mutants show defects in AFD-ending shape and, because AFD is the animal’s major thermo-sensory neuron, thermo-sensory behavior. Importantly, the degree of pruning is a function of the level of sensory input, here temperature. CED-10/Rac GTPase acts downstream of neuron activity, and CED-10/Rac GTPase expression levels dictate sensory ending engulfment rates. Interestingly, engulfment of AFD endings differs from cell corpse engulfment in that, rather than clearance being all or none (takes place, or not), it is dynamically regulated. Pruning by AMsh glia differs from phagocytosis of apoptotic corpses also in that CED-1/MEGF10 is not required and PSR-1, which plays only a minor role in apoptotic phagocytosis (Wang et al., 2003; Wang and Yang 2016), is an important regulator. The PtdSer -bridging molecule TTR-52/transthyretin, which is implicated in apoptotic phagocytosis and axon regeneration (Wang et al., 2010; Neumann et al., 2015), also regulates AFD pruning. PSR-1 and TTR-52/transthyretin function within the same pathway mediating PtdSer recognition by AMsh glia.

#### 2.5.5 Compartmentalized Cell Elimination

**FIGURE 6:**
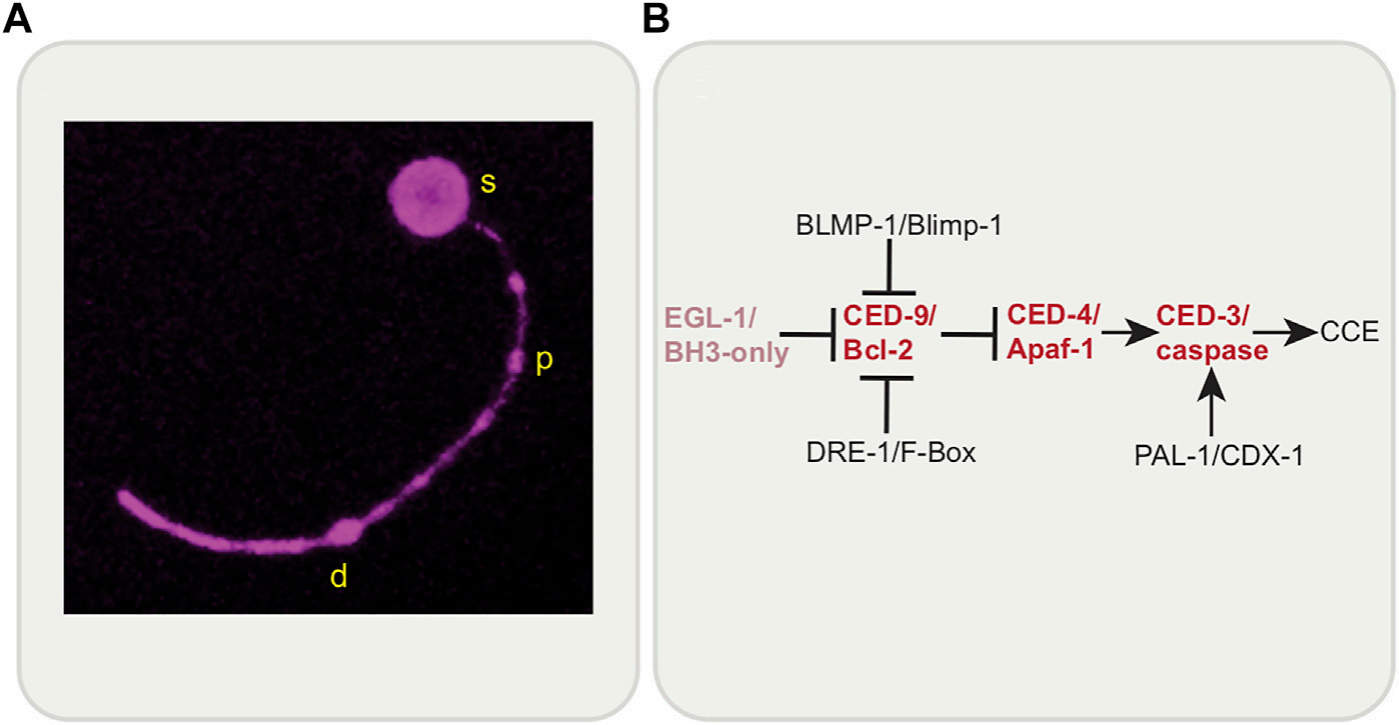
Compartmentalized Cell Elimination (CCE). **(A)** *C. elegans* tail-spike cell undergoing Compartmentalized Cell Elimination (CCE). The tail-spike cell segments into three parts; soma (s), proximal process (p), and distal process (d). Each compartment is eliminated in a CED-3/caspase-dependent manner in distinct ways; soma rounds, proximal process fragments and distal process retracts into itself. Reporter: tail-spike cell promoter-driven myristoylated mKate2. **(B)** Genetic pathway of CCE involves members of the canonical apoptosis pathway (except for EGL-1/BH3-only) and novel regulators, BLMP-1/BLIMP-1, DRE-1/F-Box and PAL-1/CDX-1.

The study of the elaborately orchestrated death of the *C. elegans* sex-specific CEM neurons (that die in the hermaphrodite embryo, living in males to function in pheromone sensing) and the tail-spike epithelial cell (which shapes the tail, and also dies in the embryo), will provide much insight into localized cell elimination. Both of these morphologically complex cells have a single process (dendrite in the case of the CEMs) and die in the same, stereotyped and likely conserved, way, a process called Compartmentalized Cell Elimination (CCE) (Ghose et al., 2018; Ghose and Shaham 2020) (Figure 6A). Genetically, death involves only some components of the core apoptotic pathway along with new regulators (Maurer et al., 2007; Chiorazzi et al., 2013; Jiang et al., 2021) (Figure 6B). Morphologically, the dying cells segment into three compartments: the soma, the proximal dendrite/process segment and the distal dendrite/process segment. Each compartment is dismantled in a CED-3/caspase-dependent manner, but in disparate ways. The soma dies as an apoptotic cell would. However, the single process/dendrite dies in two ways: the soma-proximal dendrite/process undergoes fragmentation, as in neurite pruning or Wallerian degeneration, while the distal dendrite/process undergoes retraction as seen in neurite pruning following nutrient deprivation. Therefore, dendrite/process elimination is more controlled and organized than sheer mass destruction of the cell would imply, suggesting more regulated functions of cell death genes. While CCE does require CED-3/caspase and CED-4/Apaf-1, EGL-1/BH3-only is not required. Novel regulators have been identified including PAL-1/CDX-1, DRE-1/F-Box and BLMP-1/BLIMP-1 (Maurer et al., 2007; Chiorazzi et al., 2013; Jiang et al., 2021). Novel downstream caspase targets are yet to be identified. In addition, the canonical engulfment CED-5/DOCK180 pathway is required for soma clearance but not process clearance. CCE is set apart from canonical apoptosis, given that EGL-1/BH3-only is not involved and that process elimination shows morphological hallmarks of developmental pruning. Interestingly, while a role for PtdSer in CCE per se is yet to be determined, EFF-1 fusogen promotes process elimination specifically (Ghose et al., 2018) and is required for phagosome sealing. Given what has been uncovered thus far, a study of CCE has strong potential for the discovery of novel genes in cell death and localized elimination and for new roles for cell death genes, such as CED-3/Caspase.

## 3 Discussion

Studies from other systems have shown non-apoptotic roles of cell death genes in various contexts including in the nervous system (Unsain and Barker 2015; Hollville and Deshmukh 2018). Finding such roles in the *C. elegans* nervous system is advantageous given the tractability of the nematode system and the ease with which the system may allow us to address a number of unanswered questions. For instance, studying non-canonical functions of cell death genes in the nematode nervous system may help uncover novel *in vivo* caspase protease targets as well as to better understand basic elements of nervous system development and function. The topic brings up a number of intriguing questions. How are cell elimination genes re-purposed to refine or restore as opposed to killing and ingesting? How do destructive genes regulate and calibrate their activity to allow for more controlled regressive events? The multifaceted functions of cell death players are also intriguing. How does PtdSer become an “eat-me” versus a “save-me” signal? How is EFF-1 fusogen’s function controlled such that it can perform cell-cell fusion, phagosome sealing and axonal fusion?

Studies described here bring to attention the novel regulation of the cell death machinery along with the idea of new downstream targets. As shown in axon regeneration studies, CED-3/caspase and CED-4/Apaf-1 likely have non-canonical regulators, exemplified by CRT-1/calreticulin and calcium. Control under calcium may maintain a low-level of activity thus regulating the proteolytic function of CED-3/caspase. It has also been suggested that CED-3/caspase may lie in reserve in the axon at low levels or in an inactive form which can be rapidly activated upon injury, or be rapidly translated locally, such that there is not enough global activity to allow for whole cell death but just enough to act locally. Downstream targets may negatively regulate apoptotic gene activity. For example, in mammals, gelsolin plays an anti-apoptotic role (Leifeld et al., 2006) and therefore GSNL-1 in worms may inhibit the cell death pathway that controls it from further activation and broader destruction. The studies described here also suggest novel *in vivo* caspase targets. CED-3/caspase may cleave cytoskeletal proteins to cause structural rearrangements to the axon required for filopodia extension for regeneration. It will be interesting to look at actin, tubulin and GSNL-1 as in synapse elimination, in the context of regeneration. In general, identifying caspase targets is a challenging endeavor given that caspases can be involved in the negative regulation or activation of its target. Studies of phenomena such as CCE may offer valuable insights in how cell death genes can regulate aspects of region-specific neuronal elimination.

**FIGURE 7:**
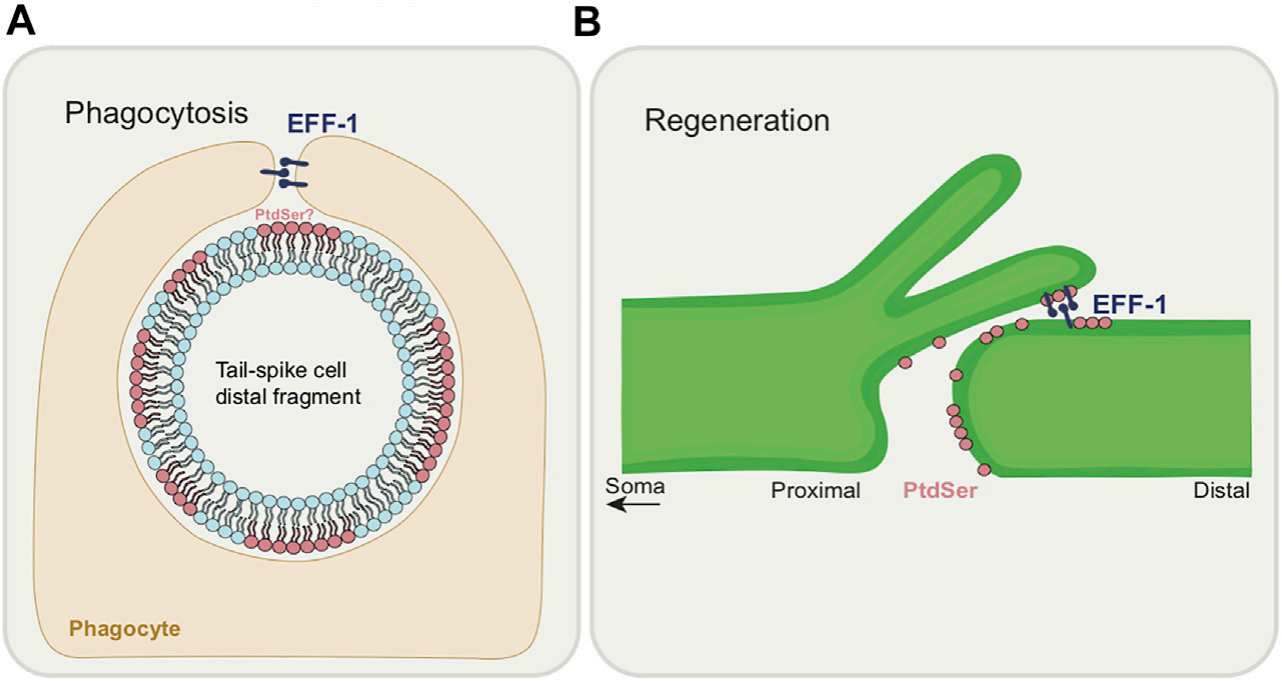
Dual functions of EFF-1 and PtdSer during phagocytosis and regeneration. **(A)** Roles of phagocytosis during CCE. EFF-1 functions in phagosome sealing during tail-spike cell distal process elimination. An involvement for PtdSer as an “eat-me” signal in CCE is yet to be demonstrated. **(B)** Roles in axon regeneration. PtdSer acts as a “save-me” signal at the severed axon membrane. EFF-1 fuses the proximal axon with the distal segment.

The roles of PtdSer and EFF-1 in phagocytosis (Figure 7A) and regeneration (Figure 7B) are noteworthy to compare. Each molecule has opposing roles, in cell destruction and in cell integrity. Each is intimately associated with membrane dynamics with PtdSer being a component of the plasma membrane and EFF-1 having the ability to fuse it. While PtdSer appears to always act cell autonomously, EFF-1 acts autonomously in its regeneration role but non-autonomously for phagocytosis. Is there a relationship between EFF-1 and PtdSer? For example, is EFF-1 driven to potential fusion sites due to the presence or absence of PtdSer on the membrane? This would be an exciting problem to resolve.

Among cell death genes, the engulfment gene CED-10/Rac GTPase has been implicated in post-embryonic neuron outgrowth and guidance during development (Gitai et al., 2003; Gabel et al., 2008). Whether other components of the cell death and engulfment machinery are involved remains to be seen. An important observation is that studies involving non-canonical roles of cell death gene in neurons have thus far been restricted largely to post-embryonic stages. It is worth noting that most somatic cell deaths take place in the embryo, with 113 cells dying out of the 131 cells that die overall in the hermaphrodite (Sulston and Horvitz 1977; Sulston et al., 1983). As such, cell death genes are very much active embryonically. Therefore, it is conceivable that many events in embryonic neurodevelopment may be linked to cell death gene function. This may include outgrowth, guidance, pruning and general nervous system assembly, for which there is much current interest. Time-lapse imaging neurons of cell death mutants across embryonic development will reveal whether obvious morphological defects occur when cell death genes are compromised. Using sparse reporters for neurons and looking at the expression pattern of cell death genes over time in the embryo will be informative. Light sheet microscopy will be an important approach to accomplish this end (Chen et al., 2014; Kumar et al., 2014).

In conclusion, as in other systems, apoptotic genes have several non-apoptotic roles in the *C. elegans* nervous system. Overall, the new horizon likely lies in neurodevelopment. The nematode system can be efficiently leveraged to better understand nervous system development by examining the role of cell death genes in embryonic neurodevelopment, and this will be an important future advance.
